# COVID-19 Vaccine Acceptance and Hesitancy among Nurses in Thailand: Implications, Challenges, and Future Prospects for Attitudes and Vaccine Literacy

**DOI:** 10.3390/vaccines12020142

**Published:** 2024-01-29

**Authors:** Nipaporn Butsing, Wantana Maneesriwongul, Poolsuk Janepanish Visudtibhan, Sirirat Leelacharas, Kamonrat Kittipimpanon

**Affiliations:** Ramathibodi School of Nursing, Faculty of Medicine Ramathibodi Hospital, Mahidol University, Bangkok 10400, Thailand; nipaporn.but@mahidol.edu (N.B.); poolsuk.jan@mahidol.ac.th (P.J.V.); sirirat.lee@mahidol.edu (S.L.); kamonrat.kit@mahidol.ac.th (K.K.)

**Keywords:** COVID-19, influenza, vaccine, vaccination, vaccine literacy, attitudes, nurses, Thailand

## Abstract

Nurses are healthcare workers at high risk of contracting COVID-19 and are prioritized for COVID-19 vaccination. This study aimed to explore COVID-19 vaccine acceptance, vaccine literacy, and attitudes toward COVID-19 vaccines, and determine factors associated with COVID-19 vaccine acceptance among nurses in Thailand. A cross-sectional survey was conducted using online questionnaires between May and June 2021. A total of 510 nurses were recruited during the pandemic’s third wave in Thailand. Data were analyzed using descriptive and inferential statistics. Ten percent (*n* = 51) of the participants were men, all of whom showed COVID-19 vaccine acceptance. Of the 459 female nurses, 94.8% (*n* = 435) accepted COVID-19 vaccination and 91.3% (*n* = 419) reported previous influenza vaccination. In multiple logistic regression models, previous influenza vaccination, interactive–critical vaccine literacy, and attitudes toward COVID-19 vaccines were significant predictors of COVID-19 vaccine acceptance among female nurses in Thailand. Those who had previously received influenza vaccination were more likely to accept COVID-19 vaccination. Higher scores for interactive–critical vaccine literacy and positive attitudes toward the COVID-19 vaccine increased the odds of accepting vaccination, while negative attitudes were associated with decreased vaccine acceptance. Vaccine literacy, together with attitudes toward the COVID-19 vaccine, had a strong positive effect on increasing vaccination acceptance and reducing vaccine hesitancy. The results suggest that policymakers should consider both attitudes and vaccine literacy when establishing prospective strategies for promoting vaccine acceptance among nurses beyond the COVID-19 pandemic.

## 1. Introduction

The coronavirus disease 2019 (COVID-19) outbreak originated in Wuhan, China, in late 2019 and was subsequently declared a pandemic in March 2020 [1]. The World Health Organization has since recommended global policies and strategies for the prevention, early detection, and medical treatment of COVID-19. These include vaccine development to limit the number of infections [2]. COVID-19 vaccines have become a vital method of combating the pandemic, as they help protect infected people from severe illness and death [3,4]. An effective COVID-19 vaccine acceptance rate is crucial for achieving herd immunity [5].

Nurses, as frontline healthcare professionals, have an increased risk of contracting COVID-19. Infection risk among nurses and other healthcare workers increased during the pandemic because of factors including exposure to infected patients, inadequate infection control, and shortages of personal protective equipment [6]. In early 2020, healthcare personnel represented 6% of adults hospitalized with COVID-19 in the United States; among them, over 36% were in nursing-related occupations [7]. During the pandemic’s early stages, a report of healthcare workers affected by COVID-19 in 37 countries revealed 58,473 nurses had been infected and about 440 had died from the disease [8]. Nurses are not only at high risk of contracting COVID-19, but they may also become carriers who transmit the virus to others. Available and effective COVID-19 vaccination is necessary for preventing transmission.

Although nurses have been prioritized for receiving COVID-19 vaccination, general vaccine acceptance rates among nurses have varied among countries [5,9,10,11,12,13,14,15]. Results from a survey of U.S. healthcare professionals in late 2020 [15] showed nurses had the lowest intention of receiving the COVID-19 vaccine (41.2%) among healthcare professionals. In early 2020, 63% of nurses in Hong Kong intended to receive the vaccine [6]. In Israel and Egypt, nurses’ intent to receive the vaccine was lower than for other healthcare workers, especially physicians [9,16], and more than half of the nurses were undecided about the COVID-19 vaccination [16]. Nurses’ acceptance rates of the COVID-19 vaccine in China (≥77%) have been stable across different time points [12,17].

In Thailand, the first COVID-19 case was identified in January 2020 [18]. From the start of the pandemic in February 2020 to November 2021, 4270 cases were reported among healthcare workers in Thailand [19]. Amid the epidemic’s third wave in Thailand, the national COVID-19 vaccination policy has prioritized frontline healthcare workers since March 2021, followed, since June 2021, by older adults and people with underlying health conditions, such as chronic respiratory diseases, cardiovascular diseases, chronic kidney diseases, cerebrovascular diseases, cancer, diabetes mellitus, and obesity [20].

Although healthcare workers have been prioritized for receiving COVID-19 vaccination, some Thai nurses have been hesitant or even refused to get vaccinated [21]. Vaccine availability does not ensure sufficient coverage to reach herd immunity, especially when healthcare workers demonstrate vaccine hesitancy [9], which has been identified as a significant obstacle to vaccine acceptance [5,9,22]. Concerns about COVID-19 vaccines’ safety and efficacy are the main reason for vaccine hesitancy [23].

The World Health Organization considers vaccine hesitancy a top-10 global health threat [24]. Vaccine hesitancy is defined as “a state of indecisiveness regarding a vaccination decision” [25] and can be driven by factors such as age [21,23,26], education [26,27], underlying diseases [21,26], and other barriers [28]. Barriers to COVID-19 vaccination can be classified into structural and attitude-related. Commonly reported structural barriers include cost, clinic location, and transportation to reach healthcare services [29]. Barriers associated with attitudes—a “person’s general feeling of being positively or negatively disposed toward a particular stimulus object” [30]—commonly include individuals’ beliefs or perceptions that reduce their willingness to be vaccinated.

Despite nurses’ clinical education, misinformation, misconceptions, and a lack of knowledge of newly developed COVID-19 vaccines have led to negative attitudes among some nurses [31]. There is a strong link between negative attitudes about vaccines and vaccine hesitancy, whereas positive attitudes have a strong positive effect on individuals’ willingness to be vaccinated [27,32]. Attitudes toward COVID-19 vaccines have been strongly regarded as a key factor influencing vaccination intention, acceptability, and hesitancy [16,26,27,32,33]. While people with positive attitudes have higher rates of vaccine acceptance, those with negative attitudes are more likely to decline or be undecided [16,34]. Such factors can be derived when applying the theory of planned behavior, through which people’s attitudes can be seen as key factors determining vaccine intention [35].

Vaccine literacy (VL) is another important factor related to vaccine acceptance and hesitancy [36,37,38]. VL refers to an individual’s skill and ability to acquire the knowledge needed for deciding to accept vaccination [38]. VL is similar to health literacy, but is more specific to understanding and communicating about vaccines [39]. As VL helps individuals to understand the rationale behind suggestions and the potential consequences of their actions [40], it is strongly associated with COVID-19 vaccine intention and hesitancy [33,36,40]. Thus, it is important for all healthcare professionals, and the public, to be “vaccine literate” [40].

No prior studies have reported the COVID-19 vaccine acceptance or hesitancy among nurses in Thailand. Accordingly, the rates and reasons for this population’s acceptance and hesitancy also have not been reported, nor have VL and attitudes toward COVID-19 vaccines among Thai nurses. To fill this knowledge gap, we conducted a cross-sectional survey of Thai nurses to investigate the acceptance rates of COVID-19 vaccines, vaccine hesitancy rates, VL, and attitudes toward the vaccines, examine associations among these factors, and determine factors associated with COVID-19 vaccine acceptance among Thai nurses.

## 2. Materials and Methods

### 2.1. Survey Population and Recruitment

Actively working nurses in Thailand were the target population. A total of 510 Thai nurses working in healthcare settings were recruited, using convenient sampling, to complete an online survey. Data were collected between May and June 2021, with two inclusion criteria: (1) registered nurses who were currently working in healthcare settings and (2) an agreement to participate in the survey. The recruitment messages containing links to the survey website and a quick response code were distributed through several social media channels, including websites, Facebook, a LINE group of individuals’ networks, and community social network groups. The participants provided informed consent online by clicking “Agree to participate in the study” before responding to the survey questions. This study was part of a larger project approved by the Committee on Human Rights Related to Research Involving Human Subjects, Faculty of Medicine Ramathibodi Hospital, Mahidol University (COA. MURA2021/381 and COA. MURA2022/45).

### 2.2. Measurements

The questionnaires covered sociodemographic characteristics, the Thai-COVID-19 VL scale, attitudes toward COVID-19 vaccines, and acceptance of COVID-19 vaccines. Participant characteristics included age, sex, region of residence, highest level of educational attainment, marital status, underlying diseases, previous influenza vaccination, and reasons for COVID-19 vaccine acceptance or vaccine hesitancy.

Acceptance of COVID-19 vaccines was measured via two questions developed by Maneesriwongul and colleagues [37]: “Have you received the COVID-19 vaccination?” (yes or no), followed by “Will you accept to receive the COVID-19 vaccine?” (will get it for sure/not sure/will not get it). Vaccine acceptance was then classified into two categories: acceptance for those who answered “yes” for the first question and responded “will get it for sure” for the second question, and hesitancy for those who answered “no” for the first question and “not sure” or “will not get it” for the second question.

COVID-19 VL was measured via the Thai COVID-19 VL scale. Biasio and colleagues [41] developed the original English version of the scale, which was translated from English to Thai [37]. The scale has 12 items classified into 2 subscales: functional VL (four items) and interactive–critical VL (eight items). The functional VL items’ wording focuses on a person’s language skills necessary to understand information, while the interactive–critical VL items address the person’s cognitive efforts, such as communication, decision-making, and problem-solving. Item responses are on a 4-point scale (from 1: never to 4: often). Four items require reverse scoring. A higher mean score indicates a higher VL level [41]. The Thai COVID-19 VL scale had adequate Cronbach’s alpha coefficients: 0.813 overall, 0.814 for the functional VL subscale, and 0.922 for the interactive–critical VL subscale.

Attitudes toward COVID-19 vaccines were assessed via a 10-item questionnaire developed by Kittipimpanon and colleagues [33]. This scale comprises five positive and five negative items. Example questions for attitudes toward the COVID-19 vaccine are “COVID-19 vaccines are safe and effective” (positive) and “COVID-19 vaccination can cause adverse effects” (negative). Each of the 10 items was rated on a 7-point scale (1: strongly disagree to 7: strongly agree). The means of five positive attitudes and five negative attitudes toward COVID-19 vaccines were separately calculated. Higher mean scores of positive or negative attitudes, respectively, indicated higher positive or negative attitudes toward the vaccination. Scale validity was verified via a previous study by experts in nursing, communicable diseases, and epidemiology [33]. The questionnaire was piloted on 40 individuals. Cronbach’s alpha for consistency reliability was 0.882 for positive attitudes and 0.724 for negative attitudes toward the COVID-19 vaccine.

### 2.3. Statistical Analysis

Descriptive statistics were performed to explore the sociodemographic variables. Frequencies and percentages were used for the categorical variables. Mean and standard deviation (SD) were used for the continuous variables. Continuous variables were further analyzed by examining skewness and kurtosis by using standardized Z values (>−1.96 and <1.96) to check for normal distribution [42]. However, we limited the inferential statistical analysis to the 459 female participants because of the low number of male participants (*n* = 51), all of whom fully accepted COVID-19 vaccination.

The differences between COVID-19 VL and attitudes toward the COVID-19 vaccine by background characteristics and vaccine acceptance were tested using an independent *t*-test or analysis of variance (ANOVA), as appropriate. The Mann–Whitney U test was used for two binary variables violating the normality assumption.

Logistic regression was used to identify factors associated with COVID-19 vaccine acceptance. Backward elimination was used to identify the multiple logistic regression model. An alpha of 0.05 was set for statistical significance. R 3.6.3 software [43] was used to perform all statistical analyses.

## 3. Results

### 3.1. Study Population Characteristics

Of the 510 nurses who completed questionnaires, the greatest number were from the central region of Thailand (32.2%), followed by Bangkok (28.8%) and the northeast region (13.9%). Most participants were female (90.0%) and ages ranged from 22 to 65 years (mean, 42.1; SD, 11.1). Over half of the female nurses were aged >40 years (58.4%), while a majority of the male nurses were younger, aged ≤40 years (66.7%). About half of the female participants had earned an education higher than a bachelor’s degree, while most male nurses had earned a bachelor’s degree as their highest level of education (60.8%). Dyslipidemia was the most prevalent underlying disease for all participants, followed by hypertension. Over 90% of participants had received an influenza vaccination. All the male nurses were receptive to COVID-19 vaccination, while 5.2% of females were hesitant. Table 1 shows more details on the participants’ characteristics.

**Figure 1:**
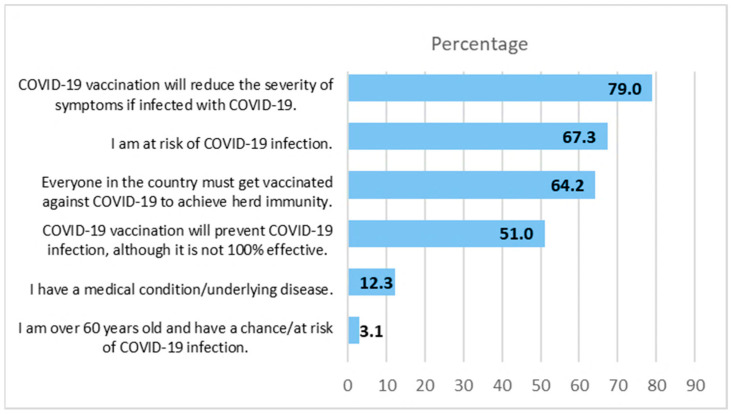
Reasons for accepting COVID-19 vaccination (*n* = 486).

Figure 1 shows the reasons for COVID-19 vaccine acceptance. The three main reasons were believing that vaccination could reduce COVID-19 symptom severity, understanding the risk of contracting COVID-19, and recognizing the need to achieve herd immunity. Table 2 shows COVID-19 vaccine hesitancy and the respective number of participants who have those reasons. The top three reasons were that the participants wanted a well-tested COVID-19 vaccine, were concerned about vaccine safety, and preferred to wait for a more effective vaccine.

### 3.2. COVID-19 Vaccine Literacy and Attitudes toward COVID-19 Vaccine

Mean functional VL, interactive–critical VL, positive attitudes, and negative attitudes toward the COVID-19 vaccine among male participants were 2.97 (SD, 0.77), 3.67 (SD, 0.31), 5.82 (SD, 1.02), and 3.97 (SD, 1.05), respectively.

Table 3 shows COVID-19 VL and attitudes toward the vaccines among the 459 female participants. Means of interactive–critical VL, positive attitudes, and negative attitudes differed significantly by age group. Older age group participants showed higher scores for positive attitude and lower scores for negative attitude compared with those in younger age groups. Higher levels of education had a significantly positive relationship with higher scores for interactive–critical VL and positive attitudes toward vaccination. Functional VL scores did not differ between participants who showed COVID-19 vaccine acceptance and those who were hesitant. However, participants who showed acceptance had higher scores for interactive–critical VL and positive attitudes, while the vaccine-hesitant participants had higher scores for negative attitudes.

### 3.3. Factors Associated with COVID-19 Vaccine Acceptance

Table 4 shows the results of logistic regression analysis (of the 459 female participants). There were four significant predictors of COVID-19 vaccine acceptance: previous influenza vaccination, interactive–critical VL, positive attitudes toward the vaccine, and negative attitudes toward the vaccine. Participants who reported previous influenza vaccination were more likely to accept the COVID-19 vaccine (adjusted odds ratio (OR) = 4.25). The odds of vaccine acceptance increased with an increased score for interactive–critical VL (adjusted OR = 2.48) and positive attitudes toward the COVID-19 vaccine (adjusted OR = 1.83). The higher the negative attitudes were toward the vaccine, the less likely the participants were to accept the vaccine (adjusted OR = 0.41). The Hosmer–Lemeshow test indicated a good logistic regression model fit (*p* = 0.476).

## 4. Discussion

The participants were nurses from all regions of Thailand (regional representation of 5.5% to 32.2%), and most were women (90.0%). This ratio aligned with the report of the Thailand Nursing and Midwifery Council in 2021 that the proportion of female registered nurses in Thailand was 94.6% [44]. The highest numbers of nurses were in the northeast, Bangkok, and central regions of Thailand [45].

Because of the low number of male participants (*n* = 51, 10%), we separately described the background characteristics and COVID-19 vaccine acceptance between male and female participants. As all the male nurses showed vaccine acceptance (had no variability in vaccine acceptance), we included only the female nurses for inferential statistical data analyses to compare their COVID-19 VL and attitudes toward the vaccine by background characteristics and vaccine acceptance, as well as logistic regression analyses, to examine factors influencing COVID-19 vaccine acceptance.

COVID-19 vaccine acceptance rates differed among countries by the time periods when data were collected, national vaccine rollouts, and specific COVID-19 situations [46]. Our study revealed a high COVID-19 vaccine acceptance rate of 95.3% among Thai nurses (94.8% for women, 100.0% for men). We report higher rates than those for nurses in other countries, which ranged widely from 24% to 61% [9,10,12,14,15,17,32]. However, the acceptance rate for Thai nurses in our study was lower than the rates reported by other healthcare personnel, including physicians, who reported the highest acceptance rates [7,9,15,47].

The reported reasons for vaccine acceptance among Thai nurses were similar to those in other reports, including fear of being infected with COVID-19, COVID-19 severity reduction, working in a high-risk environment, and the need to achieve herd immunity [9,13,48]. Vaccine mandates and social pressures were also reported [15]. Some participants reported they were induced by organizational mandates or social pressures from colleagues to receive the COVID-19 vaccines; however, the decision was theirs.

The hesitancy rate among nurses in our study was lower than those reported in the U.S. (33.6%) [15] and Hong Kong (42.9%) [17]. Other findings were also concerned with the newly developed vaccines’ efficacy and safety, as participants wanted to wait for a well-tested COVID-19 vaccine. Some nurses from previous studies have also indicated their distrust in vaccine development and testing, fear of side effects, and mistrust of the vaccine administration system [11,16,47,49]. Additionally, acceptance of and hesitancy toward COVID-19 vaccines have varied by background, beliefs, and attitudes toward vaccines [12,47].

The high rates of acceptance in our study might be due to this study’s survey being conducted 3 months after the start of Thailand’s COVID-19 vaccine rollout, which prioritized frontline healthcare workers. The data in this study were collected during the pandemic’s third wave, when COVID-19 infection and mortality rates were rising. The findings from this study were consistent with other reports on healthcare workers [14,32]. Moreover, our study population was currently working Thai nurses; thus, these nurses probably had better access to more reliable sources of information than the public, who had varying backgrounds and received information from multiple, and perhaps less reliable, sources [50,51]. However, there may be differences with other studies due to each setting’s vaccine administration and resources, including the data collection period and vaccine availability.

In our study, multiple logistic regression models, previous influenza vaccination, interactive–critical VL, and attitudes toward COVID-19 vaccines were significant factors influencing COVID-19 vaccine acceptance. Previous influenza vaccination uptake was an important predictive factor of acceptance. This finding was consistent with previous reports [52,53,54]. Our study revealed that nurses with higher interactive–critical VL had a higher chance of accepting COVID-19 vaccination. High interactive–critical VL might have led to appropriate decisions via communication with other healthcare professionals who had valid information about the vaccines. One study in Turkey [36] found negative correlations between interactive–critical VL and vaccine hesitancy in healthcare providers. There were no previous reports about VL and acceptance of the COVID-19 vaccine among healthcare workers in Thailand.

Attitudes toward COVID-19 vaccines could affect vaccine acceptance differently [23,26,27,33]. Our study revealed positive and negative attitudes toward vaccines and identified significant predictors of vaccine acceptance among nurses in Thailand. Participants who had positive attitudes were more likely to accept vaccination, while those with negative attitudes were less likely to accept vaccination. These results were consistent with findings that positive attitudes toward COVID-19 vaccines significantly increased the willingness to be vaccinated [27,55,56]. Moreover, previous studies in Egypt [57] and the Democratic Republic of the Congo [10] also found that healthcare workers’ attitudes toward COVID-19 vaccination were a main predictor of acceptance [10,57]. Additionally, attitudes toward COVID-19 vaccines were predictors of acceptance among people living in Thailand [27] and among Thai parents [58].

Our study also reported reasons for vaccine hesitancy. The main reasons for hesitancy or reluctance were vaccine safety, efficacy, side effects, and trust in vaccine development. The reasons identified from this study were consistent with those in previous studies [12,13,15,16,23,56,59,60]. A survey of physicians in Thailand [60] revealed that access to COVID-19 vaccine information was a significant predictor of COVID-19 vaccine hesitancy, and Thai physicians who accepted the vaccine were more likely to recommend it to their families or patients. Additionally, a study in Japan [59] reported that healthcare workers with lower VL were more likely to worry about the vaccines’ efficacy and future side effects, while those with higher VL were more likely to recommend COVID-19 vaccination to their clients. Thus, high VL among healthcare workers was essential, as it reinforced their own COVID-19 vaccine acceptance/intention and intention to recommend COVID-19 vaccines to others [36,59].

### 4.1. Implications, Challenges, and Future Prospects

This study revealed that VL and attitudes toward COVID-19 vaccines played important roles in heightening vaccine acceptance among Thai nurses. Uncertainty of vaccine safety and efficacy and fear of adverse side effects were major hesitancy-related concerns. These findings provide insight for policymakers to design effective communication with nurses and healthcare workers about vaccine safety and efficacy so that they fully understand that the COVID-19 vaccines’ benefits outweigh the potential risks. This will minimize negative attitudes and maximize positive attitudes. Positive attitudes among nurses and other healthcare workers could play an important role in raising public vaccine confidence and increasing COVID-19 vaccination uptake and similar uptake in the event of future pandemics. Thus, policymakers should consider both attitudes and VL when establishing prospective strategies for promoting vaccine acceptance.

### 4.2. Strengths and Limitations

This study has several strengths. It was the first study to assess COVID-19 VL, attitudes toward COVID-19 vaccines, and COVID-19 vaccine acceptance among nurses who were working in Thailand during the third wave of the COVID-19 pandemic. It was also conducted soon after the COVID-19 vaccine became available in Thailand. Additionally, the study reports on both COVID-19 vaccine acceptance and previous influenza vaccination among nurses. These findings will be useful in developing future vaccine campaigns for nurses and other healthcare workers. Finally, using the online survey format allowed us to reach Thai nurses from every region in the country.

Although the respondents represented all regions, caution should be exercised in generalizing the results because of some limitations. A key limitation was the low number of male nurses (*n* = 51, 10%), all of whom showed vaccine acceptance (had no variability in vaccine acceptance). This limited our inferential statistical analyses, as we were unable to examine factors influencing COVID-19 vaccine acceptance applicable to all nurses. Accordingly, this limited the generalizability of the findings. This study also did not collect data on working units/wards, which might influence vaccinations. Additionally, the convenience sampling used for our online survey might have introduced biases; notably, social desirability may have led to over-reporting of high acceptance rates.

## 5. Conclusions

This study revealed that previous influenza vaccination, interactive–critical VL, and positive and negative attitudes toward the COVID-19 vaccine were significant predictors of COVID-19 vaccine acceptance among nurses in Thailand. Although functional VL was not significantly related to COVID-19 vaccine acceptance, interactive-critical VL was significantly related to COVID-19 vaccine acceptance. Reasons for vaccine acceptance included being at risk of COVID-19 infection, believing the vaccine could reduce disease severity, and achieving herd immunity. Concerns about vaccine safety, efficacy, side effects, and trustworthiness of COVID-19 vaccine development were the main reasons for vaccine hesitancy.

## Figures and Tables

**Table 1:** Participants’ sociodemographic characteristics and COVID-19 vaccine acceptance (*n* = 510).

Variable	Female (*n* = 459)	Male (*n* = 51)
	*n* (%)	*n* (%)
Regions		
Bangkok	140 (30.5)	7 (13.7)
Central	136 (29.6)	22 (43.1)
North	37 (8.1)	3 (5.9)
Northeast	64 (13.9)	7 (13.7)
East	25 (5.4)	3 (5.9)
South	57 (12.4)	9 (17.6)
Age (years)		
22–30	75 (16.3)	20 (39.2)
31–40	116 (25.3)	14 (27.5)
40–50	143 (31.2)	12 (23.5)
51+	125 (27.2)	5 (9.8)
Education		
Bachelor’s degree	227 (49.5)	31 (60.8)
Higher than bachelor’s degree	232 (50.5)	20 (39.2)
Marital status		
Single	220 (47.9)	30 (58.8)
Married	203 (44.2)	19 (37.3)
Widowed, divorced, separated	36 (7.8)	2 (3.9)
Underlying diseases (multiple responses)		
None	302 (65.8)	37 (72.5)
Dyslipidemia	70 (15.3)	6 (11.8)
Hypertension	42 (9.2)	5 (9.8)
Obesity	29 (6.3)	3 (5.9)
Diabetes mellitus	16 (3.5)	5 (9.8)
Heart disease	13 (2.8)	1 (2.0)
Respiratory disease	7 (1.5)	0 (0.0)
Cancer	6 (1.3)	0 (0.0)
Cerebrovascular disease	4 (0.9)	1 (2.0)
Chronic kidney disease	2 (0.4)	0 (0.0)
Previous influenza vaccination		
Yes	419 (91.3)	47 (92.2)
No	40 (8.7)	4 (7.8)
COVID-19 vaccine acceptance		
Acceptance	435 (94.8)	51 (100.0)
Hesitancy	24 (5.2)	0 (0.0)

**Table 2:** Reasons for COVID-19 vaccine hesitancy and number of responses (*n* = 24).

Reasons	*n*
I want to get a well-tested COVID-19 vaccine.	20
COVID-19 vaccines may have serious/unsafe side effects.	19
I would like to wait for a more effective COVID-19 vaccine.	19
There is currently no COVID-19 vaccine from a manufacturer I trust/want to receive it from (I want alternatives or choices).	17
I may be allergic to the COVID-19 vaccine.	17
COVID-19 vaccines may affect the body in other ways that we don’t know.	17
COVID-19 vaccines may not be of good quality.	15
I would like to know more information about COVID-19 vaccines.	14
I don’t know how long the immunity from COVID-19 vaccination will last.	13
I am afraid of long-term effects of vaccination.	11
I am in good health and have low risk of COVID-19 infection.	10
If I get COVID-19, my symptoms are unlikely to be severe.	8
I want to get natural immunity rather than immunity from COVID-19 vaccination.	5
I want my body to have natural immunity against COVID-19.	3
The period of developing and testing the COVID-19 vaccines is too short.	3
I don’t like to inject any medications into my body.	3
I have medical conditions or underlying diseases that should not be subject to the COVID-19 vaccine.	2
I am afraid of needles and injections.	2
I think I acquired immunity from my previous COVID-19 infection.	1

**Table 3:** Participants’ COVID-19 vaccine literacy and attitudes toward COVID-19 vaccination by background characteristics and vaccine acceptance (*n* = 459).

Variable	Functional Vaccine Literacy	Interactive–Critical Vaccine Literacy	Positive Attitudes	Negative Attitudes
	Mean (SD)	Mean (SD)	Mean (SD)	Mean (SD)
All females	3.02 (0.69)	3.51 (0.47)	5.74 (0.93)	4.00 (1.09)
Age (years)				
22–30	3.01 (0.62)	3.36 (0.53)	5.10 (0.86)	4.27 (0.95)
31–40	3.07 (0.66)	3.49 (0.52)	5.79 (0.92)	4.17 (1.14)
40–50	2.99 (0.73)	3.56 (0.45)	5.90 (0.86)	3.75 (1.05)
51+	3.04 (0.69)	3.55 (0.40)	5.89 (0.89)	3.94 (1.11)
*p*-value	0.822	0.017	<0.001	0.001
Highest education level				
Bachelor’s degree	3.02 (0.70)	3.45 (0.53)	5.62 (0.95)	3.98 (1.00)
Higher than bachelor’s degree	3.04 (0.66)	3.60 (0.39)	5.85 (0.89)	4.01 (1.17)
*p*-value	0.763	<0.001 ^a^	0.004 ^a^	0.893 ^a^
COVID-19 vaccine acceptance				
Acceptance	3.03 (0.68)	3.52 (0.46)	5.79 (0.90)	3.93 (1.07)
Hesitancy	3.02 (0.77)	3.18 (0.52)	4.85 (1.06)	5.12 (0.85)
*p*-value	0.705 ^a^	<0.001 ^a^	<0.001 ^a^	<0.001 ^a^

Independent *t*-tests were used for two-group comparisons, one-way analysis of variance for more than two-group comparisons, and ^a^ *p*-value from Mann–Whitney test. SD, standard deviation.

**Table 4:** Logistic regression model predicting COVID-19 vaccine acceptance (*n* = 459).

Variable	Univariate Analysis	Multivariate Analysis
	Crude OR (95% CI)	Adjusted OR (95% CI)
Age (ref: 22–30)		
31–40	2.65 (0.83–8.44)	-
41–50	4.15 (1.21–14.27)	-
51+	2.01 (0.70–5.79)	-
Education (ref: bachelor’s degree)		
Higher than bachelor’s degree	0.86 (0.38–1.96)	-
Previous influenza vaccination (ref: no)		
Yes	3.93 (1.46–10.56)	4.25 (1.32–13.65)
Functional vaccine literacy	1.01 (0.55–1.84)	-
Interactive–critical vaccine literacy	3.06 (1.57–5.99)	2.48 (1.01–5.59)
Positive attitude toward COVID-19 vaccination	2.23 (1.56–3.19)	1.83 (1.24–2.71)
Negative attitude toward COVID-19 vaccination	0.35 (0.22–0.53)	0.41 (0.25–0.67)

OR, odds ratio; CI, confidence interval; ref, reference group; Hosmer–Lemeshow test, *p*-value = 0.476; Cox–Snell R^2^ = 0.095; Nagelkerke R^2^ = 0.282.

## Data Availability

Data supporting the reported results are available from the corresponding author upon reasonable request.
